# A Novel Adaptive Feature Fusion Strategy for Image Retrieval

**DOI:** 10.3390/e23121670

**Published:** 2021-12-12

**Authors:** Xiaojun Lu, Libo Zhang, Lei Niu, Qing Chen, Jianping Wang

**Affiliations:** College of Sciences, North Eastern University, Shenyang 110819, China; 2070038@stu.neu.edu.cn (L.Z.); 2100125@stu.neu.edu.cn (L.N.); 2070003@stu.neu.edu.cn (Q.C.); wangjianping@mail.neu.edu.cn (J.W.)

**Keywords:** image retrieval, feature fusion, pagerank, information entropy

## Abstract

In the era of big data, it is challenging to efficiently retrieve the required images from the vast amount of data. Therefore, a content-based image retrieval system is an important research direction to address this problem. Furthermore, a multi-feature-based image retrieval system can compensate for the shortage of a single feature to a certain extent, which is essential for improving retrieval system performance. Feature selection and feature fusion strategies are critical in the study of multi-feature fusion image retrieval. This paper proposes a multi-feature fusion image retrieval strategy with adaptive features based on information entropy theory. Firstly, we extract the image features, construct the distance function to calculate the similarity using the information entropy proposed in this paper, and obtain the initial retrieval results. Then, we obtain the precision of single feature retrieval based on the correlation feedback as the retrieval trust and use the retrieval trust to select the effective features automatically. After that, we initialize the weights of selected features using the average weights, construct the probability transfer matrix, and use the PageRank algorithm to update the initialized feature weights to obtain the final weights. Finally, we calculate the comprehensive similarity based on the final weights and output the detection results. This has two advantages: (1) the proposed strategy uses multiple features for image retrieval, which has better performance and more substantial generalization than the retrieval strategy based on a single feature; (2) compared with the fixed-feature retrieval strategy, our method selects the best features for fusion in each query, which takes full advantages of each feature. The experimental results show that our proposed method outperforms other methods. In the datasets of Corel1k, UC Merced Land-Use, and RSSCN7, the top10 retrieval precision is 99.55%, 88.02%, and 88.28%, respectively. In the Holidays dataset, the mean average precision (mAP) was 92.46%.

## 1. Introduction

Image retrieval aims to find images related to images of interest to users from the image dataset. Over the past 20 years, research in this area has focused on improving the accuracy and efficiency of retrieval [1]. There are two common types of image retrieval systems: text-based image retrieval systems and content-based image retrieval systems. Text-based image retrieval systems require experienced experts to tag images, which is expensive and time-consuming [2]. In contrast, content-based image retrieval uses computers to replace the manual processing of many repetitive tasks, reducing the cost of human, financial, and material resources, so content-based image retrieval is gradually becoming the mainstream method. Content-based image retrieval systems fall into two categories [3]: global features indexed with hashing strategies; local scale-invariant features indexed by a vocabulary tree or a k-d tree. Both approaches have advantages and disadvantages, and their performance complements each other [1,3].

More advanced feature extraction techniques and more appropriate feature selection and fusion strategies are crucial to improve image retrieval performance. In recent years, with the development of deep learning and convolutional neural networks, feature extraction techniques for images have improved substantially compared to the previous ones. Compared with traditional features, CNN features have more powerful representation capabilities. The authors in [4] proposed a fast multi-label feature selection method based on information theory feature ranking, which sorts features according to the importance of features, and then selects the top-ranking features. In feature fusion, fusion at the metric level is widely used, but selecting appropriate features and their corresponding weights for fusion to improve retrieval performance is still an important issue. The authors in [5] used average global weights to fuse Color and Texture features for image retrieval. The authors in [6] traverse the weight domain to select optimal weights for retrieval performance as the final weights.

Unlike previous feature selection and weight processing methods, this paper first selects valid features automatically based on the correlation feedback of single features and gives them the same initial weight; then updates the initial weights using the PageRank algorithm to obtain the final weight of each feature; finally calculates a comprehensive metric based on the final weights to output the retrieved results. For feature selection, we first extract some features and then use their retrieval accuracy as trust, then rank the trust and select some of them with higher trust for fusion. In feature fusion, we first give the same initial weights to the selected features; then construct a probability transfer matrix based on their trust; and finally update the feature weights using the PageRank algorithm, normalizing the final result obtained as the weights of each feature.

The rest of the paper is organized as follows. The second section describes some of the related work involved in this paper. The third section describes the feature extraction and processing process. The fourth section describes the feature selection and fusion strategy of this paper in detail. The fifth section presents the experimental results. The sixth section summarizes the work in this paper and gives suggestions for subsequent work.

## 2. Related Work

Image retrieval starts with creating an image database, inputting the retrieved image, calculating the similarity to the image in the image database, and finally outputting the retrieval result based on the image similarity.

Feature extraction technology is an essential factor affecting the retrieval effect. Before deep learning became popular, feature extraction methods were mainly based on features of color and texture, such as color histograms, color moments, local binary pattern (LBP) features, or some manual feature extractors such as scale invariant feature transform (SIFT), Gabor filters, generalized search trees (GIST) and histogram of oriented gradients (HOG). The development of deep learning technology has brought a significant breakthrough in image retrieval tasks, especially in feature extraction. CNN can be used to extract more representative depth features in images and fusing multiple depth features can help image retrieval tasks achieve better performance.

There has been a lot of work on fusing multiple features to improve retrieval system performance [7,8]. The relevance feedback algorithm [9] has addressed the semantic gap problem that arises in content-based image retrieval. The results obtained through relevance feedback are very similar to human understanding [10,11]. The main steps of relevance feedback are as follows: first, the retrieval system provides primary retrieval results based on the retrieval keywords provided by the user; then the user makes a judgment on the current retrieval results, which retrieval results are relevant to their retrieval needs and which retrieval results are irrelevant; and then the retrieval system will provide new retrieval results based on the feedback of users. This process is repeated until the search results meet the user’s needs.

The PageRank algorithm is a link analysis algorithm proposed by Google founders Larry Page and Sergey Brin in 1997 and is often used in the field of web search [12,13]. It creates a probability transfer matrix based on the links between web pages and continuously updates the initial probability vector based on the probability transfer matrix to obtain a stable probability vector to represent the probability distribution of users visiting each web page. There have been a lot of improved applications based on the traditional PageRank algorithm. The authors in [14] used the PageRank algorithm to enhance the Peer-Led Team Learning (PLTL) teaching method. The authors in [15] proposed a new algorithm for ranking online shops based on the PageRank algorithm. The authors in [16] proposed a new method based on PageRank to find the most influential individuals in online social networks.

In this paper, we propose a feature selection and fusion strategy based on information entropy and correlation feedback. We first extract features and preprocess the features, then construct similarity using information entropy and obtain the retrieval accuracy of each feature based on relevant feedback and use the accuracy as a trust to select features. Finally, we initialize the feature weights with the mean value, update the weights with the PageRank algorithm to obtain the comprehensive similarity, and obtain the retrieval results based on the comprehensive similarity.

## 3. Feature Extraction and Preprocess

In this paper, we extract multiple CNN features and traditional color and texture features simultaneously. The specific extraction process is as follows: for CNN features, we use the pre-trained model trained in the ImageNet dataset to extract the fc2 layer features of the original image and grayscale map, respectively. Here, fc2 refers to the features of the second fully connected layer of the network. Of course, in specific experiments, we can also extract features from other appropriate layers. Then, we stitch the extracted two parts of features to get a one-dimensional feature vector. For color and texture features, we extract color histogram features, denoted as color, LBP features, and GIST features for the original image only.

The dimensionality of tandem features is high, and noise points in image feature descriptors may reduce retrieval accuracy. In addition, using the high-dimensional image feature descriptors as a benchmark to calculate the similarity between the query image and the image in the database is time-costly and reduces retrieval efficiency. Based on the above two points, we need to reduce the dimensionality of the data features. Principal component analysis (PCA) is one of the common methods for data dimensionality reduction, which maps high-dimensional data to low-dimensional space with large variance by matrix mapping. It can reduce the dimensionality of data, reduce the computational effort, and avoid the loss of information as much as possible. In the experiments of this paper, we use the classical PCA algorithm to reduce the image features to different dimensions and compare the retrieval results of the features under different dimensions, which are described in Part 5 of the paper.

Since it takes a lot of time to calculate the similarity measure of multiple features, hash coding is needed to reduce the subsequent computation. The authors use binary to encode image features [17] as follows:

(1) $$ave\left(F_{i}\right)=\frac{\sum_{j=1}^{m}F\left(i,j\right)}{m}$$

(2) $$F\left(i,j\right)=\left\{ \begin{array}{l} 1\;\;\;\;\;\;F\left(i,j\right)\geq ave\left(F_{i}\right)\\ 0\;\;\;\;\;\;F\left(i,j\right)<ave\left(F_{i}\right) \end{array}\right.$$

where i∈{1,2,…,k} $ave\left(F_{i}\right)$ is the mean of the feature $F_{i}$, m is the dimension of the feature $F_{i}$, F(i,j) and is the j-th component of the feature $F_{i}$.

## 4. Proposed Method

### 4.1. Method Introduction

**Figure 1:**
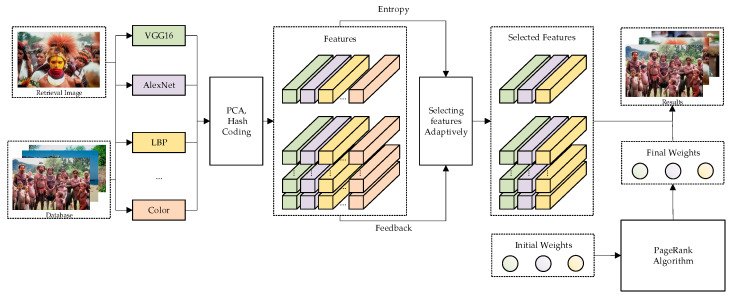
The proposed retrieval system framework.

In image retrieval methods based on multi-feature fusion, feature selection and weight determination are very important to improve retrieval accuracy. The traditional method first extracts the color, texture, or other effective features of the image and then manually selects several features for fusion. The disadvantage of this approach is that the same feature contributes differently to image retrieval in different datasets. For a given dataset, color information may be more prominent, texture information may be more prominent, or other information may be more prominent. It is difficult to determine the features suitable for the dataset and determine the optimal combination of features in advance in image retrieval work. If we try different combinations for all datasets, we can get better results, but the time consumption is vast, the efficiency is low, and the algorithm is not fixed. In response, this paper proposes a feature selection strategy using relevant feedback based on information entropy. The basic process of the retrieval system is shown in Figure 1.

As shown by Figure 1, for each retrieved image, we first extract the features of the image and process the features using PCA algorithm and hash coding; then combine information entropy and correlation feedback to select the features. Next, we initialize the feature weights using the mean value, update the weights using the PageRank algorithm. Finally, we calculate the combined similarity using selected features and final weights and output the retrieval result based on similarity.

### 4.2. Feature Selection Strategy

Information entropy is often used as a quantitative indicator of the information content of a system. In statistics, the greater the entropy, the smaller the distribution of discrimination; the smaller the entropy, the greater the discrimination of distribution. In [18], the authors used information entropy to calculate the weights of clusters, which is calculated as follows:

(3) $$H_{\left(x\right)}=\sum_{j=1}^{N}p(x)\log_{2}p{\left(x\right)}^{-1}$$

Influenced by this idea, this paper proposes a new form of information entropy to measure the differentiation in each dimension of a single feature and assign different weights to each dimension. The smaller the entropy, the greater discrimination of that feature dimension, the greater contribution to the distance calculation and the greater the weight, and vice versa. Then, we construct a weighted distance function to obtain the similarity between images.

In this paper, we use the trust obtained based on entropy and relevant feedback to select these functions. Suppose there are k extracted features, denoted as $F_{i}\left(p\right),F_{i}\left(q\right)\left(i\in \left\{1,2,\ldots ,k\right\}\right)$, q is the image to be retrieved, *p* is the image in the database. Firstly, we calculate the retrieval accuracy of a single feature based on users’ feedback on the retrieval results. Then, the retrieval accuracy is regarded as the trust of a single feature, and the trust is used to select features.

Firstly, under feature $F_{i}$, calculate the distance between the retrieved image q and all images in the database, and get the similarity $S_{i}\left(q\right)$ between them based on the distance. The calculation process of similarity $S_{i}\left(q\right)$ is:

(4) $$M^{i}\left(q\right)=\sum_{j=1}^{n}w_{j}\left|F_{q}\left(i,j\right)-F_{p}\left(i,j\right)\right|$$

(5) $$S_{i}\left(q\right)=1-\frac{1}{\sum_{q=1}^{n}M^{i}\left(q\right)}\left(M^{i}\left(1\right),M^{i}\left(2\right),\ldots ,M^{i}\left(n\right)\right)$$

where $S_{i}\left(q\right)$ is the similarity of the retrieved image q with other images in the database calculated based on the feature $F_{i}$, n is the total number of images in the image database. When calculating distances, we use Manhattan distances with weights. These weights are constructed using information entropy. $w_{j}$ is the weight of the j-th component of feature $F_{i}$, is calculated as Equations (6) and (7):

(6) $$w_{j}=\frac{e^{\left(1-h_{j}\right)}}{\sum_{j=1}^{m}e^{\left(1-h_{j}\right)}}$$

(7) $$h_{j}=-\frac{1}{\log_{2}n}\sum_{i=1}^{n}\left(\frac{f_{ij}}{\sum_{i=1}^{n}f_{ij}}\log_{2}\frac{f_{ij}}{\sum_{i=1}^{n}f_{ij}}\right)$$

here, i∈{1,2,…,n}, j∈{1,2,…,m}, n is the total number of images in the image library, m is the dimensionality of the feature, $f_{ij}$ is the j-th dimensional feature of the i-th image, and $h_{j}$ is the entropy of the j-th component of $F_{i}$. When the values of each dimension of the feature are equal, the entropy and weights of each dimension are also equal, and the resulting entropy $h_{j}$ is 1.

Then, the similarity $S_{i}\left(q\right)$ is sorted, and t samples from the image database with high similarity with the retrieved image q are returned as the retrieval results, denoted as $L^{i}=\left\{L_{1}^{i},L_{2}^{i},\ldots ,L_{t}^{i}\right\}$, where t is the predefined number of returned images. The query precision is calculated according to the retrieval results and relevant feedback. The query precision is the trust of a single feature, denoted as P(i),i∈{1,2,…,k}. In the Holidays dataset, the accuracy is measured by mAP, and in the other datasets, the accuracy is measured by precision. After the single feature trust is obtained, they are ranked from highest to lowest, and the top 5 features are selected for fusion based on trust.

### 4.3. Post-Processing of Weights by the PageRank Algorithm

Influenced by the PageRank algorithm, this paper takes the probability vector as a feature weight to measure the importance of each feature in feature fusion and uses the PageRank algorithm to obtain the final weights. Firstly, we construct the probability transfer matrix according to the retrieval trust of all selected features as the preference and then update the initial weights so that the features with higher trust can obtain higher fusion weights. Using the PageRank algorithm to update weights has the following two advantages: (1) the weight of the adaptive means that we do not need to design the weight function manually; (2) the time complexity is greatly reduced. Compared with other weight updating algorithms, the PageRank algorithm does not need the help of query results or loss function and only needs to iteratively use the probability transfer matrix, which greatly reduces the calculation amount of weight updating.

Firstly, we calculate the transfer matrix of the degree of retrieval performance preference between every single feature, denoted as $TM_{kk}=\left\{TM\left(x,y\right)\right\}$. TM(x,y) is the preference of feature x,x∈{1,2,…,k} to feature y,y∈{1,2,…,k}, which is constructed as follows:

(8) $$TM(x,y)=\frac{\left[P\left(x\right)-P\left(y\right)\right]+1}{2}$$

Then, each column $TM_{kk}=\left\{TM(x,y)\right\}$ is normalized by dividing each value in the column by the sum of the values in that column. Finally, using $TM_{kk}$ to obtain the weights of single features. The process is as following Algorithm 1:
**Algorithm 1. Post-Processing of Weights by the PageRank Algorithm** [13]**Input:** the probability transfer matrix TM, hyper-parameter of γ∈[0,1] and ε, the initial weight $w_{0}$. **Output:** the final weight $w_{d}$. 1: Initialize the weight $w_{0}=1/k$. 2: d=1 3: **repeat** 4:             $w_{d}=\gamma w_{d-1}+\left(1-\gamma \right)TMw_{d-1}$ 5:             $w_{d}=w_{d}/sum\left(w_{d}\right)$ 6:             d=d+1 7: **until** $\left\|w_{d}-w_{d-1}\right\|<\varepsilon$ 8: **Return**
*w_d_*
where $w_{0}$ is the initial weight, d refers to the number of iterations, ε,γ is the parameter, in this paper ε=0.005,γ=0.4. After post-processing by the PageRank algorithm, the final weights are used to calculate the integrated similarity measure:

(9) $$S\left(p\right)=\sum_{i=1}^{k}w_{d}^{i}S_{i}\left(q\right)$$

Finally, the retrieval results are output according to the integrated similarity metric.

## 5. Performance Evaluation

### 5.1. Datasets

To test the effectiveness of the method proposed in this paper, we conducted experiments in four image retrieval datasets: Holidays, Corel-1k, UC Merced Land-Use, and RSSCN7. The details of which are as follows.

Holidays dataset, containing 1491 holiday photos, in 500 categories, where the first image in each category is used as the retrieved image and the remaining images are the corresponding related images. The evaluation index for its retrieval performance is mAP.

Wang (Corel 1K) dataset, which contains 1000 images, is divided into ten categories: flowers, horses, buildings, beaches, etc. Each category contains 100 images. The precision of Top-N is used as the evaluation index for the retrieval system.

The UC Merced Land-Use dataset, a remote sensing dataset describing land use, was released by the University of California and includes 2100 images divided into ten categories. The image size is 256 × 256, and the pixel resolution is 30 cm. Again, we used precision as the evaluation index for the retrieval system.

The RSSCN7 dataset, the remote sensing scene dataset, contains seven categories; each category includes 400 images, each with a pixel size of 400 × 400. These images are sampled at four different scales in different seasons and weather variations. We also used precision as the evaluation index for the retrieval system.

### 5.2. Evaluation Index

The precision of the first *N* images (top-*N*) is calculated as follows:

(10) $$\mathrm{precision}=\frac{N_{r}}{N}$$

Nr refers to the number of the first N returned images that belong to the same category as the retrieved image and N refers to the total number of returned images.

The *mAP* is the mean value of average precision (*AP*) obtained from multiple queries, which is presented by:

(11) $$mAP=\frac{1}{C}\sum_{k=1}^{C}AP_{k}$$

(12) $$AP=\frac{1}{N_{r}}\sum_{i=1}^{N}\frac{i}{LOC_{i}}$$

where $AP_{k}$ refers to the $AP_{k}$ value obtained by the query of the *k*-th image, Nr the number of the first N returned images that belong to the same category as the retrieved image $LOC_{i}$ refers to the location of the i-th image in the returned image that belongs to the same category as the retrieved image. For example, if five images belonging to the same category as the query image appear at positions 1, 3, 6, 9, and 10, the *AP* is represented as follows:

(13) AP=(1/1+2/3+3/6+4/9+5/10)/5=0.62

### 5.3. Experiment Introduction

In order to prove the effectiveness of the proposed method, we conducted experiments in four datasets: Holiday, Corel-1k, UC Merced Land Use, and RSSCN7, and analyzed the experimental results. First, we obtained and compared the following three kinds of accuracy: (1) the retrieval accuracy using a single feature; (2) the accuracy obtained by selecting five features using the method in this paper and then fusing them using the average value as the weight (AVG); (3) the accuracy obtained using the feature selection and fusion strategy proposed in this paper (Ours). Then, to further prove the effectiveness of the feature selection strategy, we analyze the relationship between the retrieval accuracy of features and the number of times they are selected. After that, to further prove the effectiveness of the PageRank algorithm, we analyzed the relationship between the retrieval accuracy of features and the final weights. Finally, we give a visualization map of the retrieval results for each dataset. All the code used in this article is shared at https://github.com/JNZYBOBO/AZYBOBO.

### 5.4. Analysis of Experimental Results

#### 5.4.1. Holidays Dataset

In the Holidays dataset, we selected the first image of each category as the retrieved images, 500 images in total, and the others as image database images. Firstly, we obtained the retrieval accuracy of each feature, AVG, and ours under different dimensionality reduction results, and the results are shown in Table 1.

From Table 1, we can see the impact of different dimensions on the retrieval results. The retrieval results of each dimension are basically the same. If only the retrieval accuracy is considered, we can choose to reduce the dimension to about 300 dimensions. If the retrieval efficiency is considered, we can choose to reduce the dimension to a lower level.

The results based on single-feature retrieval show that CNN features achieve better results in single-feature image retrieval, while Color, LBP, Gist, and other color or texture features are less effective. This is because color features cannot describe the local distribution of colors and the spatial location of each color in the image; texture features are easily affected by factors such as illumination and reflection. Meanwhile, CNN features can fully consider the local and global information of the image since they are not easily affected by factors such as location and illumination and are highly resistant to noise.Different features have different effects on image retrieval results and features with stronger expressive power can be selected using the feature selection strategy proposed in this paper. Compared with any single-feature image retrieval, using our feature selection strategy can obtain more than 10 percentage points of mAP improvement. Further, using our PageRank algorithm to update the initial weights also improves the mAP by 1–2 percentage points in different dimensions.

**Figure 2:**
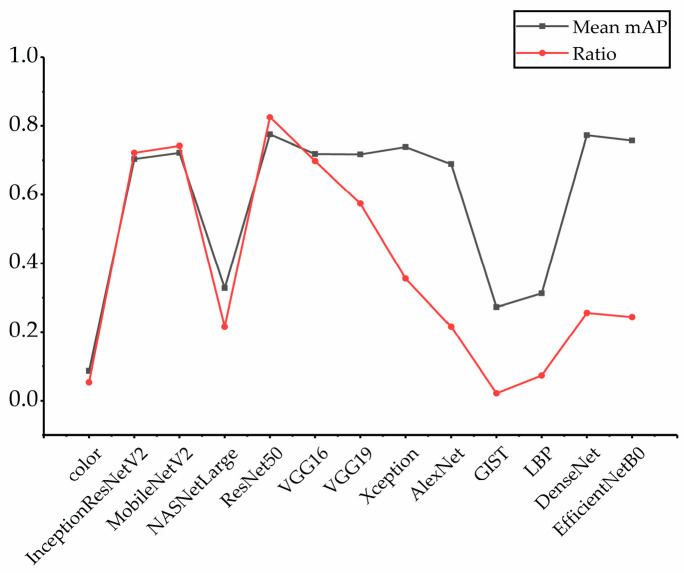
The relationship between retrieval accuracy and selected ratio.

To further prove the rationality of the feature selection strategy proposed in this paper, we counted the ratio of each feature that was selected in 500 retrieved images and compared the relationship between the retrieval accuracy of each feature and the number of times it was selected; the results are shown in Figure 2. In the figure, “Mean mAP” refers to the average value of mAPs for single feature retrieval in different dimensions in Table 1, and “Ratio” refers to the ratio of each feature selected, i.e., the number of times it is selected divided by the number of retrieved images (500). According to Figure 2, The trend of the two broken lines is approximately the same, and the higher the retrieval accuracy of the features, the higher the probability of being selected. Thus, it can be shown that our feature selection method can select features with good results.

**Figure 3:**
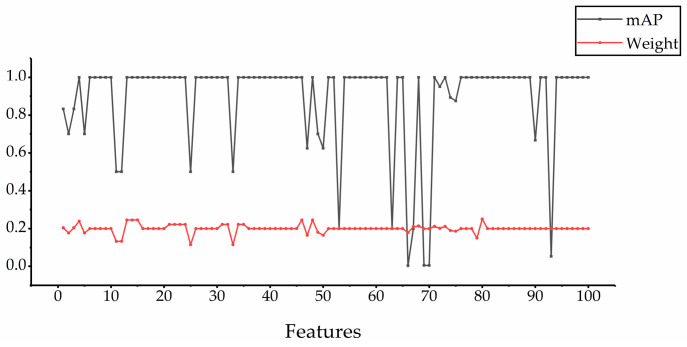
The relationship between retrieval accuracy and final weights.

In order to further prove the effectiveness of the PageRank algorithm proposed in this paper, we use 20 retrieval images to obtain the mAP of the selected five features and the final weight after updating, a total of 100 mAP values and 100 weight values. The relationship between the two is shown in Figure 3. From the figure, we can see two things: (1) the retrieval accuracy of the selected features is usually high, which further illustrates the rationality of our feature selection strategy; (2) in general, for each feature, the greater the retrieval accuracy, the greater the final weight. Thus, in feature fusion, the PageRank algorithm can give higher weight to the features with higher retrieval accuracy, so that the fused features have a better retrieval effect.

In the multi-feature fusion-based image retrieval system, compared with manual feature selection and design of weight functions, the feature selection strategy proposed in this paper can automatically select the most superior features for different images, while using the PageRank algorithm can automatically update the feature weights and adjust the weight combinations according to the feature trust. Therefore, it has higher retrieval accuracy and robustness. Table 2 shows the comparison between the experimental results in the Holidays dataset and other methods for manual selection of features and fusion weights. The optimal precision of image retrieval based on this method is 0.9246. Compared with the literature [19], retrieval accuracy increased by about 0.0126, compared with the literature [17], retrieval accuracy increased by about 0.0401, compared with the literature [20], retrieval accuracy increased by about 0.0446, compared with the literature [21], retrieval accuracy increased by about 0.0526, compared with the literature [2], retrieval accuracy increased by about 0.0556, compared with the literature [3], and retrieval accuracy increased by about 0.0694.

#### 5.4.2. Corel-1k Dataset

**Figure 4:**
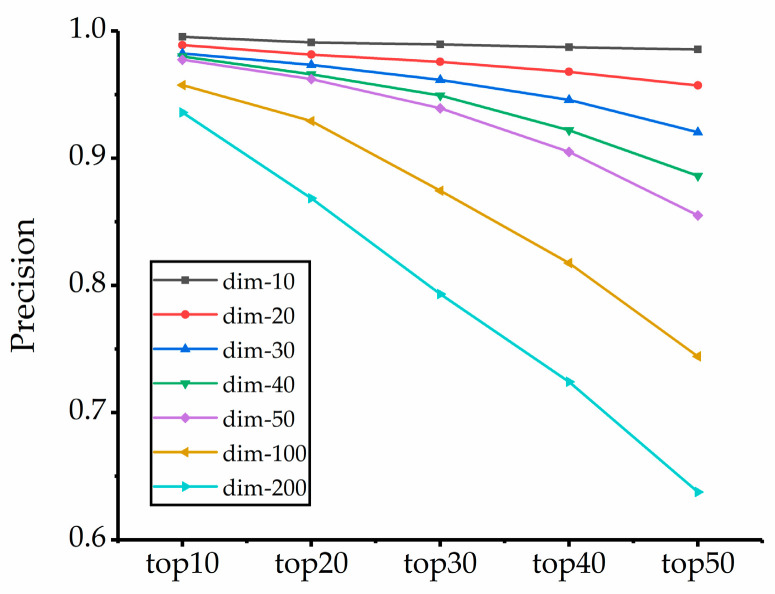
Precision in the Corel-1k dataset.

For the Corel-1K dataset, we randomly selected 20% of the images in each category as the images to be retrieved, for a total of 200 images, and the rest as the database images. Firstly, we reduce the dimensionality of each extracted feature by the PCA dimensionality reduction algorithm and then obtain the precision of top10, top20, top30, top40, and top50 in different dimensions. The results are shown in Figure 4. As we can see, image retrieval accuracy is highest when the features drop to 10 dimensions among any number of returned images. The reduction in dimensionality not only improves precision but also reduces computational effort and improves retrieval efficiency.

From the comparison of Table 1 and Table 3, we can see that the same features have different retrieval effects in different datasets, like AlexNet, ResNet50 works well in the Holidays dataset but poorly in the Corel-1k dataset, and NASNetLarge on the contrary. Furthermore, the features required for different images in the same dataset are different. According to the traditional method, we cannot determine the suitable features for different datasets or different images in the same dataset in advance. In contrast, using our feature selection strategy ensures that for each retrieved image, as long as there is a feature to be selected with better effect, it will definitely be selected, and the fused effect will definitely be better than the best retrieval effect of all features. Table 3 compares three kinds of accuracy on the different number of returned images when the feature dimension is 10. It can be seen that in different return numbers, the feature selection strategy in this paper has achieved better detection results. The detection accuracy is nearly 10 percentage points better than that of any single feature. At the same time, the use of the PageRank algorithm also makes the detection accuracy in different top indicators achieve a certain degree of improvement.

**Figure 5:**
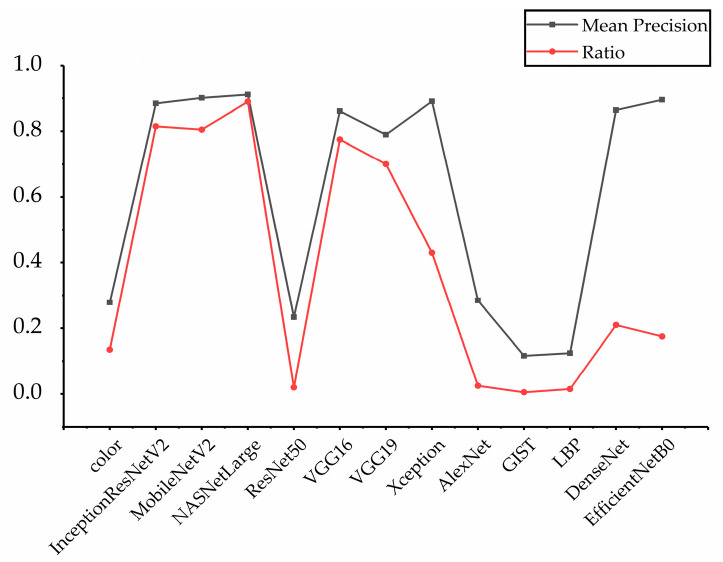
The relationship between retrieval accuracy and selected ratio.

Similarly, we also counted the ratio of each feature being selected on 200 retrieved images, and the relationship between these ratios and retrieval accuracy is shown in Figure 5. In the figure, “Mean Precision” refers to the average value of precision for single feature retrieval in different top indicators in Table 3, and “Ratio” still refers to the rate at which each feature is selected. We can see that compared with Figure 2, the trend between the two is more consistent in Figure 5, indicating that the feature selection strategy is more effective in the Corel-1K dataset, which can also be seen from the final experimental results in Table 1 and Table 3. This is because the Holidays dataset has more categories and fewer samples in each category, which are easily affected by randomness and lead to unstable results. In contrast, the Corel-1K dataset has fewer categories and a larger number of images in each category, which is more robust.

**Figure 6:**
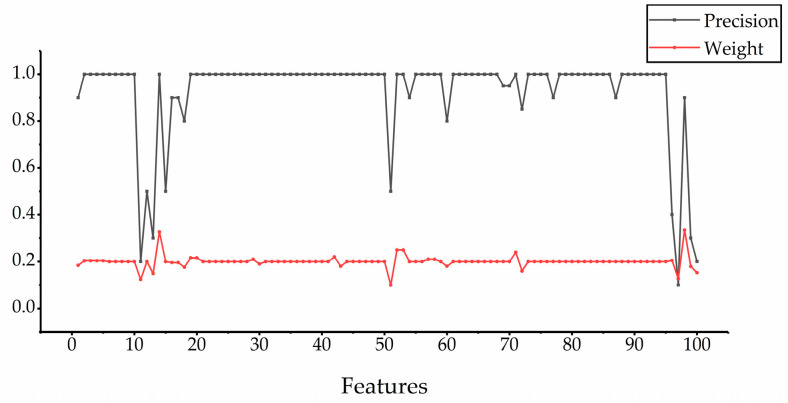
The relationship between retrieval accuracy and final weights.

Figure 6 shows the optimization effect of the PageRank algorithm on fusion weights. According to Figure 6, it can be seen that the PageRank algorithm optimizes the fusion weights according to the retrieval accuracy, giving higher weights to the excellent features to make the best final retrieval results. Compared with the Holidays dataset, the effect is more obvious in the Corel-1K dataset.

At the same time, we compared the results with the results of other papers in the Corel-1K dataset, as shown in Table 4. The accuracy of our method is 0.9955. Compared with the methods proposed in other papers, our method has obvious advantages.

#### 5.4.3. UC Merced Land Use and RSSCN7 Dataset

Similarly, we conducted experiments in the UC Merced Land Use and RSCN7 datasets. As with the Corel-1K dataset, we also used 20% of each category of images as the retrieved images and the rest as the database images. According to the results after tuning the parameters, we choose to reduce the features extracted from the RSCN7 dataset to 40 dimensions and the features extracted from the UC Merced Land Use dataset to 20 dimensions, after which we obtain the retrieval accuracy under different top metrics, and the results are shown in Table 5 and Table 6. Table 5 shows the precision on different tops in the UC Merced Land Use dataset, and Table 6 shows the precision on different tops in the RSSCN7 dataset. The detection results in both datasets also demonstrate the rationality of the proposed method in this paper. With different evaluation metrics, the detection results are greatly improved by using our method in both cases.

**Figure 7:**
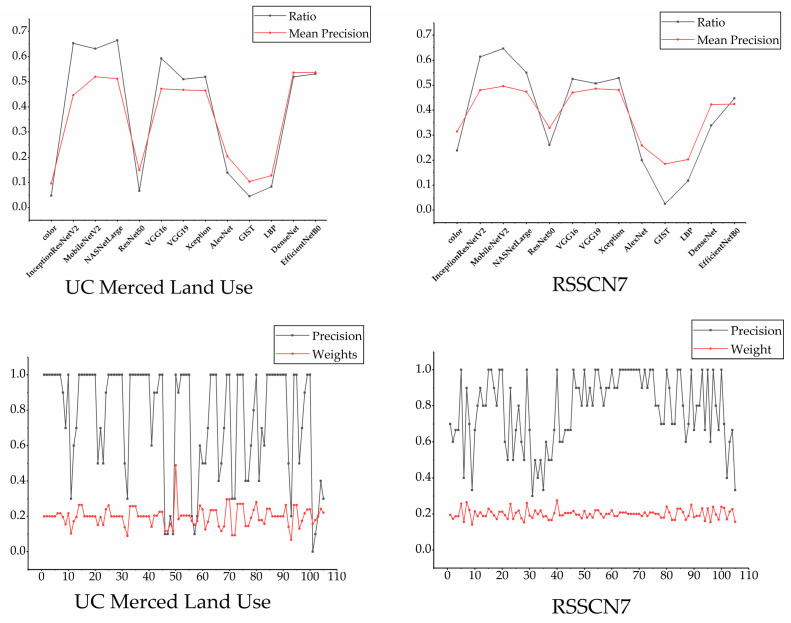
Two sets of relations in the last two datasets.

We also demonstrate the effectiveness of the feature selection strategy and weight update strategy in both datasets. In the UC Merced Land Use and RSSCN7 datasets, we selected 10 images in each category to obtain the relationship between the selected ratio and the mean precision of each feature. In addition, we selected 21 images to obtain the relationship between the retrieval accuracy and the final weights, and all the relationships are shown in Figure 7. From Figure 7, we can see that the proposed method still has good results in these two datasets.

#### 5.4.4. Visualization Result

Lastly, in order to make the experimental results clearer, we conducted tests in all datasets to visualize the retrieval results returned, as shown in Table 7. In the “Results and similarity” column, images with green borders are correct results, images with red borders are incorrect results, and the numbers below the images are their similarity to the retrieved images. In the Holidays database, because only two images are correct results of retrieval image, we used mAP to calculate the precision, and the precision was 1.0. In other databases, we used top-10 to calculate the precision; the results were 1.0, 1.0, and 0.9.

## 6. Conclusions

The multi-feature fusion strategy can improve the accuracy and generalization ability of the retrieval system. Suitable feature selection strategies and feature fusion strategies can help enhance the retrieval performance of the retrieval system ulteriorly. In this paper, we use information entropy and relevant feedback to automatically select features, use the PageRank method to process a single feature’s weight, obtain the comprehensive similarity according to the weight after processing, and finally output the retrieval results. Experimental results in the Holidays, Corel-1k, UC Merced Land-Use, and RSSCN7 show that the proposed method has better retrieval performance than single feature image retrieval systems and image retrieval systems without the PageRank algorithm. The experiments in the Holidays and Corel-1k datasets show that the proposed method is better than the existing methods. The next step is to investigate how to select the appropriate parameters and improve the retrieval performance after the features have been selected.

## Figures and Tables

**Table 1:** Retrieval performance comparison in the Holidays dataset.

Features	Dimensions								
	500	400	350	300	250	200	150	100	50
Color	0.0848	0.0874	0.0876	0.0871	0.0869	0.0870	0.0870	0.0890	0.0857
InceptionResNetV2	0.7092	0.7342	0.7306	0.7322	0.7324	0.7238	0.7121	0.6842	0.5762
MobileNetV2	0.7199	0.7357	0.7468	0.7527	0.7459	0.7414	0.7289	0.7086	0.6175
NASNetLarge	0.3109	0.3232	0.3305	0.3302	0.3392	0.3525	0.3497	0.3378	0.2774
ResNet50	0.7820	0.7903	0.7936	0.8013	0.8046	0.7953	0.7908	0.7533	0.6672
VGG16	0.7128	0.7384	0.7432	0.7553	0.7508	0.7333	0.7295	0.6991	0.6046
VGG19	0.7107	0.7444	0.7489	0.7495	0.7567	0.7406	0.7244	0.6833	0.5987
Xception	0.7401	0.7566	0.7637	0.7623	0.7696	0.7589	0.7511	0.7082	0.6355
AlexNet	0.6808	0.7007	0.7116	0.7241	0.7212	0.7172	0.7007	0.6681	0.5805
GIST	0.2552	0.2640	0.2804	0.2778	0.2909	0.2841	0.2923	0.2773	0.2324
LBP	0.3244	0.3244	0.3244	0.3244	0.3244	0.3272	0.3124	0.2909	0.2617
DenseNet	0.7548	0.7847	0.7963	0.8047	0.8047	0.7960	0.7851	0.7598	0.6687
EfficientNetB0	0.7613	0.7740	0.7850	0.7873	0.7944	0.7795	0.7680	0.7364	0.6299
AVG	0.9018	0.9028	0.9114	0.9170	0.9132	0.9143	0.9125	0.9075	0.8981
Ours	0.9194	0.9188	0.9246	0.9245	0.9178	0.9239	0.9216	0.9150	0.9050

**Table 2:** Comparison of retrieval results in the Holidays dataset.

Method	Ours	[19]	[17]	[20]	[21]	[2]	[3]
mAP	0.9246	0.9120	0.8845	0.8800	0.8720	0.8690	0.8552

**Table 3:** Precision for different numbers of returned images in the Corel-1k dataset.

Features	Top10	Top20	Top30	Top40	Top50
Color	0.2995	0.2840	0.2788	0.2686	0.2613
InceptionResNetV2	0.9115	0.9007	0.8823	0.8709	0.8570
MobileNetV2	0.9175	0.9117	0.9040	0.8949	0.8795
NASNetLarge	0.9335	0.9255	0.9115	0.9061	0.8814
ResNet50	0.2310	0.2482	0.2363	0.2226	0.2315
VGG16	0.8970	0.8777	0.8593	0.8436	0.8278
VGG19	0.8785	0.8697	0.5493	0.8364	0.8140
Xception	0.9155	0.9135	0.8993	0.8775	0.8495
AlexNet	0.2847	0.2847	0.2847	0.2847	0.2847
GIST	0.1162	0.1162	0.1162	0.1162	0.1162
LBP	0.1245	0.1245	0.1245	0.1245	0.1245
DenseNet	0.8642	0.8642	0.8642	0.8642	0.8642
EfficientNetB0	0.8957	0.8957	0.8957	0.8957	0.8957
AVG	0.9935	0.9890	0.9876	0.9861	0.9851
Ours	0.9955	0.9910	0.9895	0.9872	0.9856

**Table 4:** Comparison of search results in the Corel-1k dataset.

Method	Ours	[17]	[22]	[23]	[24]	[25]	[26]
Precision	0.9955	0.9709	0.8650	0.8550	0.8250	0.8040	0.8000

**Table 5:** Precision for different numbers of returned images in the UC Merced Land Use dataset.

Features	Top10	Top20	Top30	Top40	Top50
Color	0.1102	0.0996	0.0937	0.0879	0.0876
InceptionResNetV2	0.5369	0.4825	0.4372	0.4032	0.3744
MobileNetV2	0.6152	0.5582	0.5084	0.4747	0.4417
NASNetLarge	0.6026	0.5499	0.5035	0.4677	0.4355
ResNet50	0.1740	0.1579	0.1438	0.1356	0.1308
VGG16	0.5552	0.5015	0.4600	0.4347	0.4095
VGG19	0.5480	0.5008	0.4586	0.4292	0.4035
Xception	0.5588	0.4997	0.4533	0.4224	0.3930
AlexNet	0.2038	0.2038	0.2038	0.2038	0.2038
GIST	0.1033	0.1033	0.1033	0.1033	0.1033
LBP	0.1269	0.1269	0.1269	0.1269	0.1269
DenseNet	0.5359	0.5359	0.5359	0.5359	0.5359
EfficientNetB0	0.5369	0.5369	0.5369	0.5369	0.5369
AVG	0.8683	0.8117	0.7773	0.7368	0.6989
Ours	0.8802	0.8334	0.7879	0.7447	0.7067

**Table 6:** Precision for different numbers of returned images in the RSSCN7 dataset.

Features	Top10	Top20	Top30	Top40	Top50
Color	0.3019	0.3323	0.3253	0.3113	0.3026
InceptionResNetV2	0.5391	0.5011	0.4739	0.4509	0.4370
MobileNetV2	0.5623	0.5189	0.4877	0.4649	0.4495
NASNetLarge	0.5369	0.5025	0.4763	0.4461	0.4114
ResNet50	0.3566	0.3390	0.3285	0.3226	0.3015
VGG16	0.5316	0.4932	0.4608	0.4436	0.4278
VGG19	0.5417	0.5063	0.4848	0.4664	0.4340
Xception	0.5353	0.4984	0.4667	0.4575	0.4495
AlexNet	0.2586	0.2586	0.2586	0.2586	0.2586
GIST	0.1829	0.1829	0.1829	0.1892	0.1892
LBP	0.2031	0.2031	0.2031	0.2031	0.2031
DenseNet	0.4225	0.4225	0.4225	0.4225	0.4225
EfficientNetB0	0.4244	0.4244	0.4244	0.4244	0.4244
AVG	0.8651	0.8083	0.7683	0.7291	0.6851
Ours	0.8828	0.8326	0.7838	0.7372	0.6856

**Table 7:** Examples of image retrieval in different databases.

Database	Holidays	Corel1k	UC Merced Land Use	RSSCN7
Retrieval image	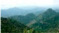	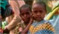	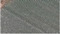	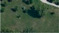
Results and similarity	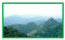 0.99952937	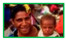 0.99975951	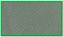 0.99985633	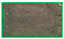 0.9997895
	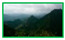 0.99932636	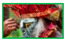 0.99974828	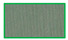 0.99983305	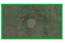 0.99977838
	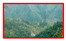 0.99932212	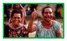 0.99974674	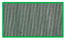 0.99983272	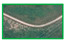 0.99976673
	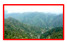 0.99931696	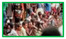 0.99970117	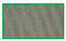 0.99982084	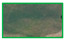 0.99976315
	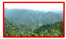 0.9992626	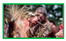 0.99969666	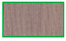 0.99982064	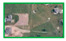 0.99976234
	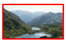 0.99917911	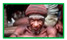 0.99969415	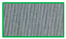 0.99982037	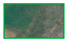 0.99975622
	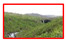 0.99915044	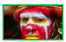 0.99965442	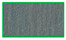 0.99982006	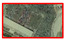 0.99975168
	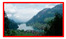 0.99913248	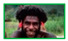 0.99965017	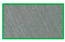 0.99980933	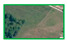 0.99975145
	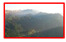 0.99911954	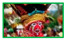 0.99964863	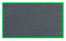 0.99980879	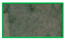 0.99975058
	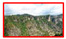 0.99909944	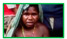 0.99964755	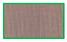 0.9998086	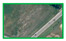 0.99974986

## Data Availability

The data is contained within the article.
